# Glycosaminoglycans from *Litopenaeus vannamei* Inhibit the Alzheimer’s Disease β Secretase, BACE1

**DOI:** 10.3390/md19040203

**Published:** 2021-04-03

**Authors:** Courtney J. Mycroft-West, Anthony J. Devlin, Lynsay C. Cooper, Scott E. Guimond, Patricia Procter, Marco Guerrini, Gavin J. Miller, David G. Fernig, Edwin A. Yates, Marcelo A. Lima, Mark A. Skidmore

**Affiliations:** 1 Molecular & Structural Biosciences, School of Life Sciences, Keele University, Huxley Building, Keele, Staffordshire ST5 5BG, UK; c.j.mycroft-west@keele.ac.uk (C.J.M.-W.); a.devlin1@keele.ac.uk (A.J.D.); l.c.cooper@keele.ac.uk (L.C.C.); p.procter@keele.ac.uk (P.P.); m.andrade.de.lima@keele.ac.uk (M.A.L.); 2 School of Medicine, Keele University, Huxley Building, Keele, Staffordshire ST5 5BG, UK; s.e.guimond@keele.ac.uk; 3 Istituto di Ricerche Chimiche e Biochimiche G. Ronzoni, via G. Colombo 81, 20133 Milan, Italy; guerrini@ronzoni.it; 4 School of Chemistry, Keele University, Huxley Building, Keele, Staffordshire ST5 5BG, UK; g.j.miller@keele.ac.uk; 5 Department of Biochemistry and Systems Biology, ISMIB, University of Liverpool, Crown Street, Liverpool L69 7ZB, UK; dgfernig@liverpool.ac.uk (D.G.F.); E.A.Yates@liverpool.ac.uk (E.A.Y.)

**Keywords:** Alzheimer’s disease, amyloid-β, BACE1, β-secretase, glycosaminoglycan, chondroitin sulfate, heparin, heparan sulphate, *Litopenaeus vannamei*

## Abstract

Only palliative therapeutic options exist for the treatment of Alzheimer’s Disease; no new successful drug candidates have been developed in over 15 years. The widely used clinical anticoagulant heparin has been reported to exert beneficial effects through multiple pathophysiological pathways involved in the aetiology of Alzheimer’s Disease, for example, amyloid peptide production and clearance, tau phosphorylation, inflammation and oxidative stress. Despite the therapeutic potential of heparin as a multi-target drug for Alzheimer’s disease, the repurposing of pharmaceutical heparin is proscribed owing to the potent anticoagulant activity of this drug. Here, a heterogenous non-anticoagulant glycosaminoglycan extract, obtained from the shrimp *Litopenaeus vannamei,* was found to inhibit the key neuronal β-secretase, BACE1, displaying a more favorable therapeutic ratio compared to pharmaceutical heparin when anticoagulant activity is considered.

## 1. Introduction

Alzheimer’s disease (AD) is a progressive, irreversible, neurodegenerative disorder characterized by the formation of extracellular amyloid plaques and intracellular neurofibrillary tangles (NFTs) within the brain. Despite many hundreds of clinical trials for novel treatment options against AD, none have delivered a viable pharmaceutical since 2003 [1] and currently only solely palliative drugs are licensed for use [2]. Hence, there is an urgent need for new therapeutic agents that can interact with the underpinning mechanisms of AD.

Amyloid deposits within the cerebral cortex are considered to be a consequence of the production of amyloidogenic amyloid precursor protein (APP) fragments generated by the sequential proteolytic processing of the type 1 transmembrane protein APP. Fragmentation of APP into amyloidogenic peptides occurs through the action of several proteases beginning with cleavage by the primary neuronal β-secretase, BACE-1, which yields a soluble N-terminal fragment (sAPPβ) and a membrane located C-terminus fragment (CTF-99). The CTF-99 fragment is further cleaved by γ-secretase, yielding Aβ peptides (predominantly Aβ 40–42) [3,4,5]. Aggregation of Aβ peptides into toxic species is thought to initiate other neuropathies such as NFTs, inflammation and oxidative stress, which together result in neuronal death, brain atrophy and AD [2]. Inhibition of the key protease that leads to the generation of Aβ fragments, BACE1, has therefore emerged as a key drug target in the development of novel AD pharmaceuticals [6].

The glycosaminoglycan (GAG) heparan sulphate (HS) has previously been identified as the physiological regulator of BACE-1 by Scholefield et al. (2003) [7] and the extant pharmaceutical anticoagulant heparin, a highly sulphated variant of HS, inhibits BACE-1 in vitro [7]. Heparin binding has been proposed to occur within a region of BACE-1 that prevents substrate access to the active site, and to act via a mechanism that differs from conventional small molecule inhibitors [7,8,9]. Glycosaminoglycan-derived oligosaccharides have also been shown to cross the blood–brain barrier [10], whilst co-localizing with and inhibiting, BACE-1 [7,11].

In addition to BACE-1 inhibition, exogenous heparin also exerts manifold beneficial effects against other AD related neuropathies. Examples include preventing Aβ proinflammatory activity, acting as a Cu^2+^ and Zn^2+^ chelator, decreasing NFT formation, and reducing apolipoprotein E associated toxicity [12,13]. Indicating that heparin and related polysaccharides have the potential to act as multi-target drugs; an approach which is considered favourable for AD drug development [14]. Mouse models treated with low-molecular weight heparin (LWMH) derivatives have also displayed decreased Aβ deposition and improved cognitive functions [15,16], providing further promise for the application of heparin as a treatment for AD. Despite this, the repurposing of mammalian-derived heparin and low molecular weight oligosaccharides as a treatment for AD is largely prohibited by the likely side effect of anticoagulation.

The GAGs HS and heparin are composed of a repeating disaccharide backbone of D-glucuronic acid (GlcA) linked β(1→4) to d-glucosamine. The repeating disaccharide backbone can possess variable levels of modifications, including epimerisation of β d-GlcA to α l-iduronic acid (IdoA) and sulphation at defined positions alone the polysaccharide chain. In addition to HS/heparin, the GAG family also contains chondroitin sulphate (CS), dermatan sulphate (DS), hyaluronic acid (HA) and keratan sulphate (KS). Chondroitin sulphate and DS both comprise a uronic acid linked β (1→3) to N-acetyl d-galactosamine, with the uronic acid residue being β d-GlcA or α L-IdoA, respectively. Chondroitin sulphate and DS can also contain varying levels of sulphate modifications [17]. The fine structure imparted by these modifications modulate protein-GAG interactions and bestow functional activities, such as BACE-1 inhibition, upon these polysaccharides [11,18]. 

In contrast to mammalian-derived GAGs, marine organisms provide a rich and diverse source of GAGs that possess increased structural diversity [9,19,20,21,22,23,24,25,26]. Glycosaminoglycans have been identified belonging to phyla such as *Cnidaria, Crustacea, Tunicata* and *Mollusca*, that are structurally related to their mammalian-derived counterparts. Regarding the constituent disaccharide units, however, they differ in their detailed disaccharide composition according to the species from which they have been extracted [27,28]. The exploration of the structural diversity offered by marine derived GAG offers increased sequence space for the discovery of GAGs with favourable bioactivities and reduced off-target effects such as anticoagulant activity [17,19,22,29]. Marine derived GAGs are additionally advantageous since they may be acquired from aquaculture waste, making their exploitation economically, environmentally and socially appealing.

Previously, we have shown HS and CS extracts obtained from the marine organisms *Portunus pelagicus* and *Sardina pilchardus* inhibit BACE1 [9,30]. These marine-derived GAGs retain favourable inhibitory activities against BACE-1, when compared to mammalian-derived heparin, whilst exhibiting diminished anticoagulation activity. Here, we report a further GAG extract obtained from *Litopenaeus vannamei,* composed of a mixture of chondroitin sulphate and heparan sulphate, that can inhibit BACE-1 and also displays attenuated anticoagulant properties.

## 2. Results

### 2.1. Isolation of Glycosaminoglycans from Litopenaeus vannamei

**Figure 1:**
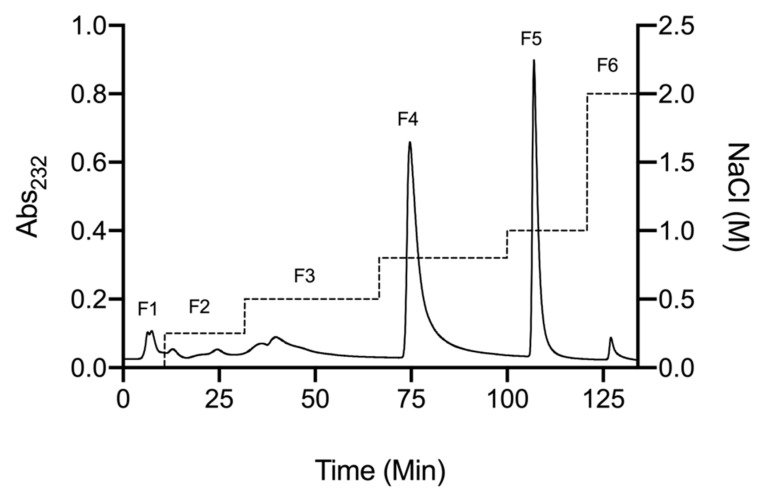
DEAE anion exchange chromatography purification of crude glycosaminoglycan from *L. vannamei*. Fractions 1–6 (solid line) correspond to a stepwise NaCl elution with in-line monitoring at 232 nm (dashed line).

A crude glycosaminoglycan extract was obtained from defatted *Litopenaeus vannamei* tissue by proteolytic digestion (Alcalase^®^; Novozymes, Krogshøjvej, Bagsvaerd, Denmark), followed by capture and elution from strongly basic anion exchange resin (Amberlite^®^ IRA-900; Sigma-Aldrich, Dorset, UK), as previously described by Mycroft-West et al. (2019 and 2020) [9,30]. The crude GAG extract was further fractionated using DEAE-based, anion-exchange chromatography employing a stepwise sodium chloride gradient for elution (Figure 1). Fractions corresponding to elution at 0.8 M (fraction 4) and 1M (fraction 5) NaCl accounted for ~43% and ~30% of the crude sample, respectively. 

The structural characteristics of *L. vannamei* fractions 4 and 5 (F4 and F5, respectively) were subsequently characterised by several spectroscopic techniques that are widely employed in the composition analysis of GAGs.

### 2.2. Characterisation of Extracted Glycosaminoglycans from Litopenaeus vannamei

#### 2.2.1. Agarose-Based, Gel Electrophoresis

**Figure 2:**
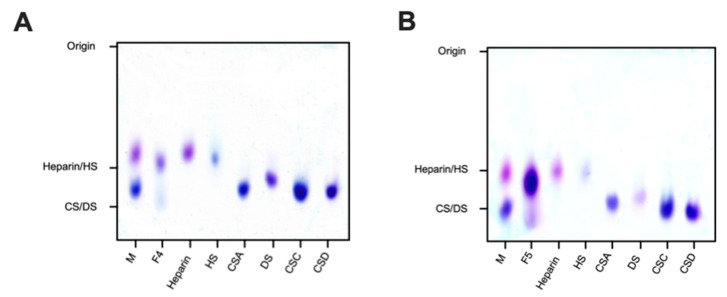
Electrophoretic mobility of *L. vannamei F4* (**A**) and F5 (**B**) and reference glycosaminoglycans; heparin, heparan sulphate (HS), dermatan sulphate (DS) and chondroitin sulphate A, C and D (CSA, CSC and CSD, respectively), M = mixture of CSA and heparin.

Agarose-based gel electrophoresis in 1,3-diaminopropane buffer (pH 9.0) was first utilised to investigate the electrophoretic mobility of *L. vannamei* F4 and F5 in comparison to GAG standards (Figure 2). Both fractions separated into two distinct bands corresponding to HS and CS, suggesting that *L. vannamei* F4 and F5 are composed of a heterogenous mixture of GAGs. The major constituent of both fractions possessed similar electrophoretic mobility to mammalian HS, migrating further than porcine heparin, but less than both CS and DS standards. A further minor band was also present in both fractions, migrating at a slightly greater distance than mono- and disulphated CS standards (Figure 2).

#### 2.2.2. Attenuated Total Reflectance Fourier Transform Infrared Spectroscopy

**Figure 3:**
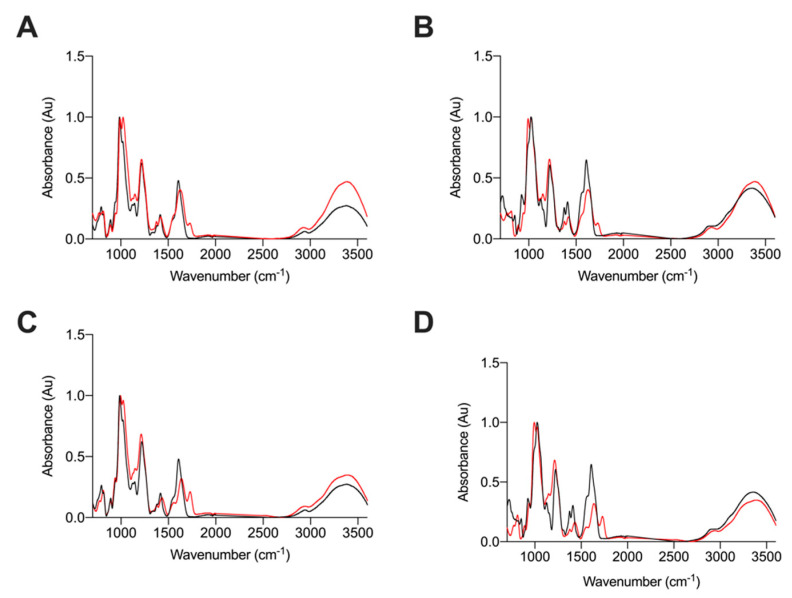
ATR-FTIR spectra of (**A**) *L. vannamei* F4 (red) and HS (black) (**B**) *L. vannamei* F4 (red) and CS (black), (**C**) *L. vannamei* F5 (red) and HS (black) and (**D**) *L. vannamei* F5 (red) and CS (black); n = 5.

As agarose gel electrophoresis revealed bands with migration distances corresponding to HS and CS, within both fractions, ATR-FTIR spectroscopy was employed to elucidate the GAG composition of *L. vannamei* F4 and F5. The ATR-FTIR spectra of *L. vannamei* F4 and F5 were compared to those of CS and HS as these were the major GAGs with corresponding migrations observed by agarose gel electrophoresis (Figure 3). Both *L. vannamei* F4 and F5 contained spectral features representative of GAGs, with peaks corresponding to the common motifs; S=O, symmetric carbonyl stretching and asymmetric stretching at 1230, 1430 and 1635 cm^−1^, respectively (Figure 3). The peak shoulder observed at 1559 cm^−1^, which is present in all samples, has also previously been assigned to the amide II band corresponding to coupled C-N vibrations of N-acetyl (amide) groups, a characteristic structural feature.

Notably, the ATR-FTIR spectra of HS exhibited a peak at 990 cm^−1^ with a peak shoulder at 1025 cm^−1^. This was reversed in the CS ATR-FTIR spectra with the main peak occurring at 1025 cm^−1^ and peak shoulder at 990 cm^−1^ (Figure 3). Bands in the region of 1200–900 cm^−1^ have previously been assigned to the C-O-C glycosidic bond stretches [31,32,33,34], therefore the differences observed between CS and HS in this region could be attributed to differences in glycosidic bond linkages between these GAGs.

*L. vannamei* F4 contained a split peak at 990 cm^−1^ and 1025 cm^−1^, whereas the peak at 990 cm^−1^ was more prominent in *L. vannamei* F5, with a peak shoulder occurring at 1025 cm^−1^. This suggests that *L. vannamei* F5 contains a higher proportion of HS than *L. vannamei* F4. There are also differences in the intensities of peaks at ~1450 cm^−1^ and 1600 cm^−1^, between HS and CS samples (Figure 3), with both L. vannamei F4 and F5 more closely resembling HS in these regions. In addition, the peak shoulder at ~1370 cm^−1^, present in both *L. vannamei* F4 and F5, has also been suggested as indicative of a HS/CS mixture [31], further supporting the notion that both fractions are composed of a mixture of these GAGs. An additional peak at ~1725 cm^−1^ was observed in both *L. vannamei* F4 and F5, but was absent in both HS and CS (Figure 3), suggesting that the *L. vannamei* GAG extracts may contain rare structural features. 

The differences between the spectra of HS, CS and *L. vannamei* F4 and F5 in the region >3000 cm^−1^ (OH stretch region) are associated with changeable moisture levels introduced during sample acquisition and not likely to result from underlying structural differences as mentioned previously [31]. 

Owing to the complex nature of GAGs and the heterogeneity of sample preparations, ATR-FTIR spectra of GAGs are often complex, with extensive signal overlap and therefore, lack the spectral resolution of 2-dimensional (^1^H-^13^C) NMR; as a result, it is not currently possible to provide complete assignment for the ATR-FTIR spectra of GAGs. Therefore, the ATR-FTIR spectra of *L. vannamei* F4 and F5 were subjected to multivariate analysis, post-acquisition, with comparison to a library of known GAGs comprising 185 heparins, 31 HS, 44 CSs and DSs, 11 HAs and 6 OSCSs, using principal component analysis (PCA).

**Figure 4:**
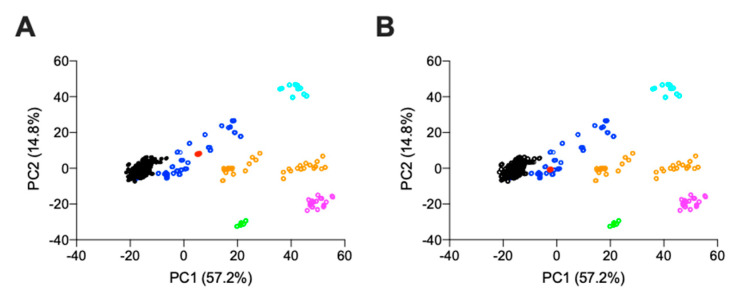
Principal component analysis score plot for PC2 vs. PC1 from the ATR-FTIR spectra of (**A**) *L. vannamei* F4 and (**B**) *L. vannamei* F5 against a bone fide GAG library. Heparin (black), HS (dark blue), CS (orange), DS (magenta), HA (cyan), over-sulphated CS (light green) and L. vannamei fractions (red).

Comparison of PC1 and PC2, which covers > 70% of the total variance, indicates that both *L. vannamei* F4 and F5 locate within the region containing mammalian HS (Figure 4A and Figure 4B, respectively). Principal component 1, which covers 57% of the total variance, indicates that both *L. vannamei* F4 and F5 separate towards the region containing CS. This region has previously been identified with heparin samples containing CS contaminants [31]. The separation towards this region was more pronounced for *L. vannamei* F4 than F5, suggesting a greater proportion of CS in F4 (Figure 4).

#### 2.2.3. Nuclear Magnetic Resonance

**Figure 5:**
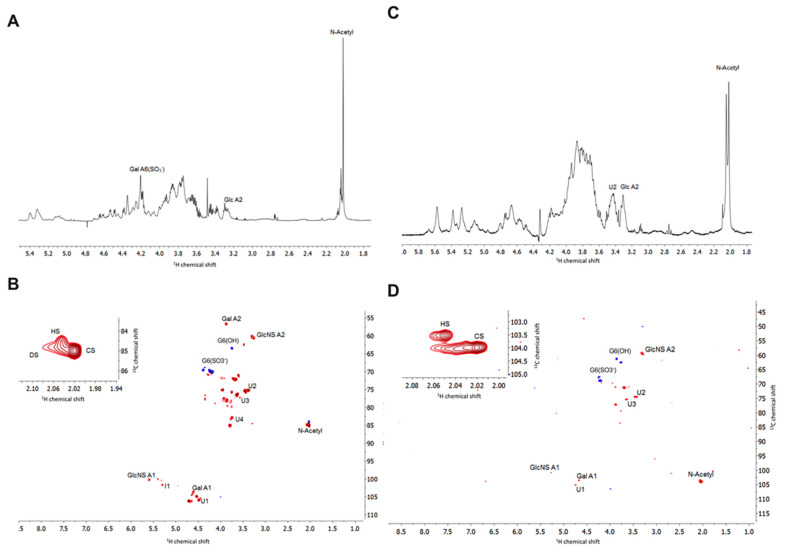
(**A**) ^1^H and (**B**) ^1^H-^13^C HSQC NMR spectra of L. vannamei F4, (**C**) 1H and (**D**) ^1^H-^13^C HSQC of *L. vannamei* F5. Major signals associated with CS and HS are indicated. Spectral integration was performed on the HSQC using labelled signals. Glucosamine, Glc; galactosamine, Gal; uronic acid, U; iduronic acid, I.

Proton and heteronuclear single-quantum correlation (HSQC) NMR spectroscopy were employed to confirm the GAG composition of *L. vannamei* F4 and F5. Through their characteristic chemical shift positions, ^1^H NMR can distinguish the major signals associated with HS, as well as those that arise from galactosaminoglycans such as CS and DS. Signals associated with the N-acetyl of CS (~2.02 ppm), DS (~2.07 ppm) and HS (~2.04 ppm) were present in both *L. vannamei F4* and F5 (Figure 5). The major N-acetyl signal in both fractions at 2.02 ppm suggest that the fractions contain a larger quantity of CS than indicated by ATR-FTIR spectroscopy and agarose gel analysis. Owing to the heterogeneity of the sample and resulting overlapping resonances in the ^1^H NMR spectra, further assignment was preformed utilising ^1^H -^13^C HSQC NMR. The signals attributed to N-acetyl signals in the ^1^H-^13^C HSQC further indicated that *L. vannamei* F4 and F5 are composed of CS, HS and DS (Figure 5, insert).

The anomeric region can be used to further distinguish GAG classes, with the resonance of the anomeric carbon of glucosamine occurring downfield to that of galactosamine. The presence of the signal at 4.5–6/103 ppm and 5.4/100 ppm in the ^1^H-^13^C HSQC of *L. vannamei* F4 and F5 can be attributed to position 1 of galactosamine and glucosamine, respectively [35]. By definition, DS is distinguished from CS through the epimerisation of the GlcA residue to IdoA. The anomeric peak of IdoA is located further downfield to that of GlcA at 5.0/105 ppm and 4.5–4.7/106 ppm, respectively [35]. A peak can be observed corresponding to the presence of GlcA, but not IdoA within the ^1^H -^13^C HSQC of *L. vannamei* F4 and F5 (Figure 5).

Within the ^1^H-^13^C HSQC spectrum of *L. vannamei* F4 several signals can be observed that can be attributed to the anomeric position of GlcA at 4.45/106–4.48/106.5ppm and 4.65/106.5–4.7/106.5 ppm, whereas for *L. vannamei* F5, one peak at 4.7/106.5 ppm is observed (Figure 5). The downfield shift in the ^1^H dimension has previously been shown to be indicative of varying levels of 2-and 3-O sulphation of GlcA residue [24,26], thereby indicating that the CS component of *L. vannamei* F4 and F5 could potentially harbour rare sulphation patterns. A low intensity peak corresponding to iduronic acid can be observed at 5.2/101 ppm in both *L. vannamei* F4 and F5, further indicating the presence of HS/heparin within both fractions. The peak at 3.4/60 ppm is attributable to position 2 of N-sulphated glucosamine and the intensity of this peak indicates that the HS component of *L. vannamei* F4 and F5 is largely N-sulphated (Figure 5). 

The peak at ~3.4/75 ppm in the ^1^H-^13^C HSQC of *L. vannamei* F4 and F5 is attributable to position 2 of GlcA [36], again additional peaks with a slight downfield shift can be observed possibly indicating the presence of sulphate modifications. Peaks at ~3.8–3.7/62–61 ppm and ~4.3–4.2/68–67ppm can be attributed G6(OH) and G6(S) of galactosamine and glucosamine, respectively. Intensities of these signals indicated that both the CS and HS component of *L. vannamei* F4 and F5 contain a higher proportion of G6(S) than G6(OH).

#### 2.2.4. Strong Anion-Exchange High Performance Liquid Chromatography Disaccharide Compositional Analysis

Since NMR spectroscopy revealed that *L. vannamei* F4 and F5 contained a significantly high proportion of CS both fractions were subjected to exhaustive digestion with chondroitinase ABC and compositional SAX-HPLC disaccharide analysis was subsequently performed. Only minimal digestion by chondroitinase ABC was observed for both *L. vannamei* fractions; evidenced by the low intensity of the HPLC chromatogram at 232 nm (A1). This could further indicate the presence of rare sulphate modifications on GlcA residues as previous reports have shown that such modifications can prohibit cleavage by chondroitinase ABC and, where digestion has been reported, the resulting products were not detectable by HPLC analysis, potentially owing to the destruction of the 3-O-sulphate modification [37,38,39]. This implies the presence of sulphate modifications within F4 and F5 that render the GAG either chain largely resistant to chondroitinase ABC digestion, resulting in oligosaccharides, and/or the presence of high levels of 3-O-sulphated GlcA residues. Matched chondroitinase ABC digestions of CSC and CSA standards were also employed as a positive control to confirm enzyme activity (A2). Digestion of CSC and CSA generated major disaccharide products bearing 6S or 4S modifications, respectively, which were detected at high intensity at 232 nm, indicating that a lack of enzymatic activity could not account for the minimal digestion of *L. vannamei* F4 and F5. The detected digest products of *L. vannamei* F4 and F5 are presented in Table 1, however, it should be emphasised that the composition reported is based only on the digestible material, which likely forms a small fraction of the sample. Having said this, the CS disaccharide composition analysis indicated that *L. vannamei* F4 possess both CSC and CSA units while, *L. vannamei* F5 possess CSC and CSE units.

*Litopenaeus vannamei* F4 and F5 were also subjected to exhaustive, enzymatic digestion with *Pedobacter heparinus* lyases I, II and III and the digestion products were subject to SAX-HPLC disaccharide composition analysis by comparing retention times to those of eight common HS/heparin Δ-disaccharide reference standards [40]. Mammalian heparin and HS controls were also extensively digested to confirm that enzymatic activity resulted in the expected digestion products, because non-mammalian GAGs have been previously reported to resist heparinase digestion [41] (Table 2).

The digestion products for both mammalian heparin and HS agreed with previously published disaccharide profiles [40]. The trisulphated, Δ-UA(2S)-GlcNS(6S) accounts for the major disaccharide present in heparin at 51.4% in comparison to HS, where this disaccharide accounts for ~7% of the total chain. As expected, mammalian HS is composed primarily of di- and mono- sulphated disaccharide standards; Δ-UA-GlcNS (17.8%), Δ-UA-GlcNAc(6S) (12.8%), Δ-UA-GlcNS(6S) and (18.0%), accounting for ~ 50% of the total detected disaccharides. This can be attributable to the NA/NS and NS-domains of HS. The second most prevalent disaccharide present in mammalian heparin was found to be Δ-UA-GlcNS(6S) (18.2%), as reported [40]. In comparison and as expected, the most prevalent disaccharide detected in the mammalian HS sample was the unsulphated, Δ-UA-GlcNAc (37.7%), which forms the primary constituent disaccharide of NA-domains within HS (Table 2).

The lyase digestion products of *L. vannamei* F4 and F5 exhibit a more varied sulphation profile than mammalian heparin and HS. Both *L. vannamei* F4 and F5 contain a lower proportion of the tri-sulphated disaccharide, Δ-UA(2S)-GlcNS(6S), when compared to heparin at ~15%, however, this is approximately double that typically present in mammalian HS (Table 1). *L. vannamei* F4 exhibited a slightly higher proportion of un-sulphated disaccharides (Δ-UA-GlcNAc) when compared to mammalian heparin at 14.8%. This is substantially lower than typically reported for HS [40,42,43]. In comparison *L. vannamei* F5 contained approximately the same proportion of Δ-UA-GlcNAc as mammalian heparin at 9.4%. Furthermore, both fractions contained a low proportion of both the trisulphated disaccharide, Δ-UA(2S)-GlcNS(6S) and unsulphated disaccharide, Δ-UA-GlcNAc, which are characteristic of heparin and HS, respectively, indicating that the *L. vannamei* fractions contain a heparin-HS like GAG with structural characteristics that are distinct to their mammalian counterparts.

Both *L. vannamei* F4 and F5 are comprised primarily of di-sulphated disaccharides which account for ~56% and 61% of the chain, respectively; the most prevalent disaccharide being Δ-UA-GlcNS(6S) for both fractions. Δ-UA-GlcNS(6S) is also the most prevalent di-sulphated disaccharide present in mammalian heparin and HS. *L. vannamei* F4 and F5 also contain ~14% mono-sulphated disaccharides of which the majority can be attributed to Δ-UA-GlcNS in both fractions, however, this disaccharide was more prevalent in *L. vannamei* F4 than F5. *L. vannamei* F5 also contains a higher proportion of the disaccharide Δ-UA-GlcAc(6S) compared to F4, at 5.7% and 3.7%, respectively. The percentage of mono-sulphated disaccharides in the *L. vannamei* fractions, more closely resembled heparin than HS which contain ~12% and 31%, respectively. The majority of the HS component of the *L. vannamei* fractions contain sulphation at position C6 of glucosamine (F4; 51% and F5; 69%) and are N-sulphated (F4; 80% and F5; 83%) rather than N-acetylated. This analysis was further confirmed by the ^1^H-^13^C NMR spectra of *L. vannamei* F4 and F5 (Figure 5).

### 2.3. Glycosaminoglycans from Litopenaeus vannamei Inhibit β-Secretase 1

**Figure 6:**
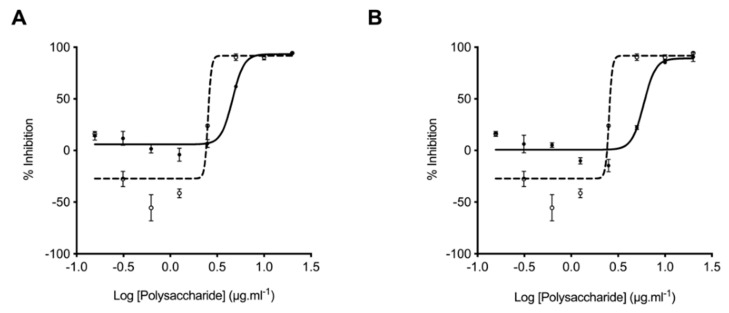
Inhibition of the human β-secretase, BACE-1. *L. vannamei* F4 (**A**) and F5 (**B**) were assayed for their ability to inhibit the human BACE-1 cleavage of a quenched fluorogenic peptide based on the Swedish mutation. Fluorescent emission was monitored for 90 min (λ_ex_ = 320 nm, λ_em_ = 405 nm). Data are presented as % inhibition calculated from the mean of the substrate only and containing no inhibitor controls ± SD (n = 3). The IC_50_ of *L. vannamei* F4 (A; solid line, filled circles) and F5 (B; solid line, filled circles) was determined to be 4.61 μg·mL^−1^ (R2 = 0.97) and 5.93 μg·mL^−1^ (R^2^ = 0.93), respectively. In contrast the IC_50_ heparin (A and B dashed line, open circles) was ~2.43 μg·mL^−1^ (R^2^ = 0.93).

The inhibition of BACE1 by *L. vannamei* F4 and F5 was determined through the deployment of the FRET peptide assay based on the APP Swedish mutation, as previously described by Mycroft-West et al. (2019 and 2020) [9,30]. In accordance with previous reports, the IC_50_ of heparin for BACE-1 inhibition was determined to be ~2 μg·mL^−1^, with maximal inhibition being achieved at concentrations greater than 5 μg·mL^−1^ [9]. Maximal BACE-1 inhibition by *L. vannamei* F4 and F5 was observed to occur at two-fold the concentration of heparin (10 μg·mL^−1^) with an IC_50_ of ~4.6 and 5.9 μg·mL^−1^, respectively. Both *L. vannamei* fractions therefore, displayed BACE1 inhibitory activity and F4 was the more potent. As previously reported [9,44] at low concentrations of heparin (1.3–0.3 μg·mL^−1^) an increase in BACE1 activity was observed (Figure 6). The extracts obtained from *L. vannamei* also promoted BACE1 activity, however this was reduced in comparison to the heparin at all concentrations tested (Figure 6).

### 2.4. Glycosaminoglycans from Litopenaeus vannamei Possess Attenuated Anticoagulant Activities

**Figure 7:**
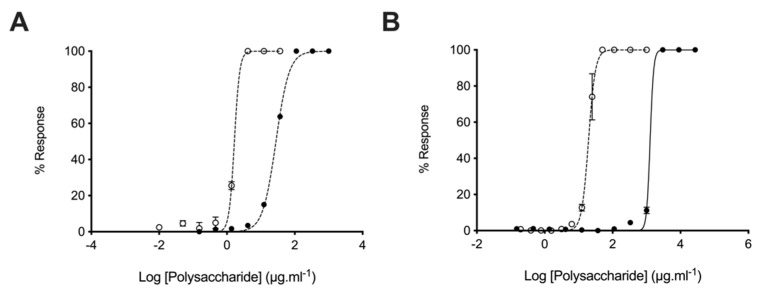
Anticoagulant activity of *L. vannamei* F4. (**A**) Activated partial thromboplastin time (aPTT) and (**B**) prothrombin time (PT) inhibitory response (mean % response, ± SD, n = 3) for heparin (open circle, dashed line) and *L. vannamei* F4 (closed circle, solid line). aPTT: heparin EC_50_ = 1.66 μg·mL^–1^; *L. vannamei* F4 EC_50_ = 27.91 μg·mL^–1^. PT: heparin EC_50_ = 19.53 μg·mL^–1^; *L. vannamei* F4 EC_50_ = 1276 μg·mL^–1^.

**Figure 8:**
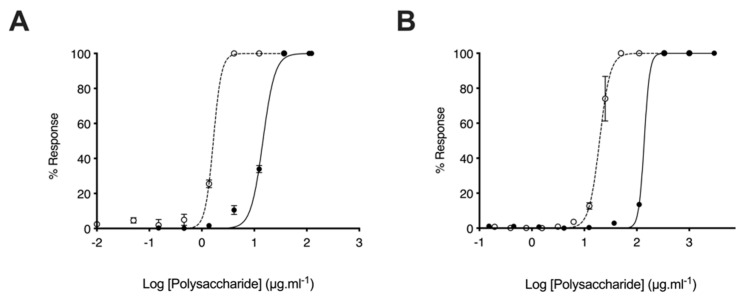
Anticoagulant activity of *L. vannamei* F5. (**A**) Activated partial thromboplastin time (aPTT) and (**B**) prothrombin time (PT) inhibitory response (mean % response, ± SD, n = 3) for heparin (open circle, dashed line) and *L. vannamei* F5 (closed circle, solid line). aPTT: heparin EC_50_ = 1.66 μg × mL^–1^; *L. vannamei* F5 EC_50_ = 14.60 μg·mL^–1^. PT: heparin EC_50_ = 19.53 μg·mL^–1^; *L. vannamei* F5 EC_50_ = 138.80 μg·mL^–1^.

The anticoagulant activity of heparin hampers the repurposing of this clinically approved drug as a therapeutic for AD. Owing to this, the anticoagulant activities of the *L. vannamei* GAG extracts were determined using the prothrombin time (PT) and activated partial thromboplastin time (aPTT) assays. In comparison to heparin (193 IU.mg^−1^), *L. vannamei* F4 displayed negligible anticoagulant activity by the PT assay, which measures the extrinsic coagulation pathway, with EC_50_ = 1276 μg·mL^−1^ and = 19.53 μg·mL^−1^, respectively (Figure 7). *L. vannamei* F5 demonstrated increased activity in the PT assay compared to F4 at EC_50_ = 138.80 μg·mL^−1^ (Figure 8), however, that is still drastically attenuated in comparison to heparin. Inhibition of the intrinsic coagulation assay was also measured by the aPTT assay, where F4 displayed >10-fold reduction in activity in comparison to heparin, at 27.91 μg·mL^−1^ and 1.66 μg·mL^–1^, respectively (Figure 7). Again, *L. vannamei* F5 displayed increased activity in the aPTT assay in comparison to *L. vannamei* F4 at EC_50_ = 14.60 μg·mL^–1^, however, the activity of both fractions was negligible compared to heparin.

Despite the reduced BACE1 inhibitory activity of F4 and F5 in comparison to heparin, when anticoagulant activity is considered *L. vannamei* F4 and F5 displayed increased therapeutic ratios of 6.10, 2.46 and 0.68, respectively (FRET:aPTT; Table 3). *L. vannamei* F4, displayed the greatest therapeutic value of the compounds tested, and as a result, was utilised for further experiments.

### 2.5. Glycosaminoglycans from Litopenaeus vannamei Decrease the Thermal Stability of BACE1

**Figure 9:**
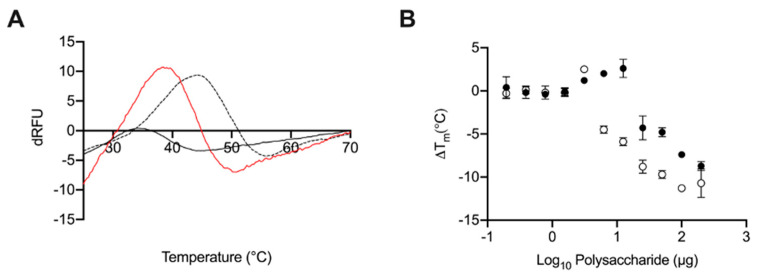
(**A**) First differential of the DSF thermal stability profile of BACE1 alone (1 µg; dashed line) and with heparin (2 μg black line) or L. vannamei F4 (2 µg red line) in 50 mM sodium acetate buffer pH 4.0. (**B**) ΔT_m_ of BACE1 with increasing heparin (open circles) or *L. vannamei F4* (closed circles) concentration.

Previously, GAGs have been shown to destabilise BACE1; observed by a reduction in the melting temperature (T_m_) of BACE1 in differential scanning fluorimetry (DSF) measurements. At 50 μg·mL^−1^, where maximal destabilisation was observed, heparin induced a ΔT_m_ of ~10 °C. At an equivalent concentration *L. vannamei* F4 induced ~5 °C reduction in T_m_ indicating that both fractions destabilised BACE1 in a similar manner to heparin and other GAGs [9,30]. Destabilisation of BACE1, as measured through DSF, by heparin and *L. vannamei* F4 was also found to be dose dependent (Figure 9).

### 2.6. Circular Dichroism Spectroscopy of Human β-Secretase (BACE-1) with and without L. vannamei F4

**Figure 10:**
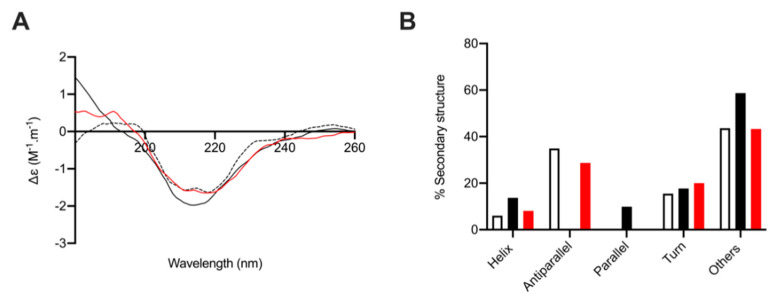
The secondary structural change of BACE1 observed in the presence of heparin and *L. vannamei F4*. (**A**) CD spectra of BACE1 alone (9 µg; dashed line) and in the presence of 36 µg heparin (black solid line) or *L. vannamei F4* (red) in 50 mM Sodium acetate buffer pH 4.0. (**B**) Secondary structure (%) of BACE1 alone (white) and in the presence of heparin (black solid line) or *L. vannamei* F4 (red), estimated using BeStSel [45] between 180–260 nm.

As reported previously, the secondary structure composition of BACE1 at pH 4.0, calculated from fitting the CD spectra against a representative protein library (BeStSel; [45]) in line with the percentage structural composition, predicated by the x-ray crystal structure of BACE1 at pH 4.0 (PDB: 2ZHS; [9,46]). The CD spectrum of BACE1 alone exhibited secondary structure composition of 6% helix, 35% antiparallel, 0% parallel, 16% turn, and 43% other, in accordance with previous reports [9]. Circular dichroism spectroscopy was then performed at 1:4 (*w*/*w*) ratio of BACE1 to polysaccharide; the same ratio at which *L. vannamei* F4 exhibited maximal inhibition in FRET assays. Under these conditions we previously reported that heparin and HS-like polysaccharides induce a conformational change in BACE1 that resulted in increased α-helix and reduced antiparallel β-sheet content [9]. In agreement with our previous reports, the CD spectra of BACE1 in the presence of heparin (1:4 *w*/*w*; BACE1:heparin) resulted in an increase in α-helix content (+8%), a reduction in antiparallel β-sheet (−35%). BACE1 in the presences of *L. vannamei* F4 (1:4 w/w; BACE1: *L. vannamei* F4) also exhibited an increase in α-helix content (+2%) and reduced antiparallel β-sheet (−6%), indicating a common conformational change in BACE1 upon GAG binding (Figure 10).

## 3. Discussion

The extract obtained from *L. vannamei* was confirmed to be composed of a heterogenous mixture of GAGs, including CS, HS and DS, by NMR spectroscopy. ^1^H-^13^C HSQC NMR revealed that *L. vannamei* F4 and F5, eluted from DEAE-Sephacel with 0.8 M and 1M NaCl, respectively were composed primarily of HS, CS and a smaller proportion of DS. When analysed by agarose gel electrophoresis, both *L. vannamei* fractions were observed to contain a major band with a migration distance corresponding to heparin/HS and a minor band corresponding to mono- and di-sulphated CS. Previous reports have indicated that CS obtained from *L. vannamei,* eluted form DEAE-Sephacel at 0.8 and 1M NaCl, also possessed similar electrophoretic mobility to HS [24,26]. The difference in electrophoretic mobility of these CS extractions from *L. vannamei* could be attributed to the presence of rare sulphate modifications at positions 2 and 3 on the GlcA residue. In support of this, in the present study ^1^H-^13^C HSQC NMR of the *L. vannamei* fractions revealed a peak at approximately 4.7/106 ppm, which was indicative of 2 or 3-sulphated GlcA residues in the aforementioned CS extractions obtained from *L. vannamei* by Palhares et al. (2019) [24] and Cavalcante et al. (2018) [26]. The presence of sulphated GlcA residues could therefore account for the higher proportion of CS within the *L. vannamei* fractions, when analysed by NMR spectroscopy, in comparison to ATR-FTIR spectroscopy and agarose electrophoresis. In further support of the presence of 2 and/or 3-sulphated GlcA modifications on the *L. vannamei* GAG fractions described in this study, digestion by chondroitinase ABC yielded minimal detectable disaccharide products when analysed by SAX-HPLC. It has previously been established that sulphate modifications on GlcA residues of chondroitin sulphate can render disaccharides resistant to chondroitinase ABC digestion [37], and GlcA-Gal disaccharides bearing 3-O sulphate modifications are largely undetectable by HPLC analysis [38]. 

In addition to sulphated GlcA residues, NMR analysis revealed that *L. vannamei* F4 and F5 contains primarily Gal6(S). The higher level of sulphation at position 6 of galactosamine in fraction 4 was also detected through SAX-HPLC disaccharide analysis of the digested components. In addition to Gal6S, SAX-HPLC disaccharide analysis revealed the presence of Gal4S (CSA) in *L. vannamei* F4 and Gal4S,6S (CSE) in *L. vannamei* F5. This is in contrast to NMR analysis, which did not detect sulphation at position 4 of galactosamine in either fraction. As digestion of both *L. vannamei* by chondroitinase ABC yielded minimal detectable disaccharide products, the absence of Gal4S in ^1^H-^13^C HSQC NMR analysis may be explained by a low incidence of this modification within the extract. Having said this, constitutional analysis by both ^1^H-^13^C HSQC NMR and SAX-HPLC reveals that the sulphation pattern of the CS component of the *L. vannamei* fractions is complex and further analysis would be required to resolve the fine structure of the CS within this extract. 

*L. vannamei* F4 and F5 possess BACE1 inhibitory activity, albeit at reduced levels in comparison to heparin, using the previously described FRET-peptide assay. *L. vannamei* F4 possesses a slightly increased BACE1 inhibitory activity in comparison to *L. vannamei F5,* with IC_50_ of 4.6 μg·mL^–1^ and 5.9 μg·mL^–1^, respectively. Both *L. vannamei* fractions are predominantly composed of a mixture of CS and HS. Previously, we have identified a CS extract from *Sardina pilchardus* [30] that displayed BACE1 inhibitory activity, therefore it cannot be assumed that HS is the sole active component of the *L. vannamei* extract and it is likely that defined sulphation patterns within CS and HS chains are required for BACE-1 inhibitory activity, as previously described [11,30]. The majority of the HS component of the *L. vannamei* fractions contained sulphation at position C6 of glucosamine (F4; 51% and F5; 69%) and N-sulphation as opposed to N-acetylation (F4; 80% and F5; 83%). Previously 6-O sulphation of heparin has been reported to be important for BACE1 inhibitory activity, therefore this extract provides further support that 6-O-sulphation of HS/heparin is important for BACE1 inhibitory activity [11]. The ability of the *L. vannamei* extract to promote proBACE1 at low concentrations was considerably diminished compared to heparin. This has also been demonstrated for other marine GAGs [8,30], indicating that this source of GAGs may be more favourable for therapeutic applications against AD.

As the anticoagulant activity of heparin-based pharmaceuticals hampers the repurposing of these clinically approved drugs for the treatment of AD, the effect of *L. vannamei* F4 and F5 on the intrinsic, extrinsic and common coagulation pathways was examined. Both *L. vannamei* fractions exhibited negligible anticoagulant activities in the aPTT and PT assays therefore, despite the diminished BACE1 inhibitory activity of *L. vannamei* F4 and F5 in comparison to heparin, the therapeutic value is greater when off target anticoagulation activity is taken into account. *L. vannamei* F4 demonstrated a higher therapeutic value than both heparin and *L. vannamei* F5.

To assess whether *L. vannamei* F4 inhibited BACE1 via a mechanism similar to heparin, the interaction with BACE1 was examined via CD spectroscopy and DSF. Circular dichroism spectroscopy of BACE1 in the presence of GAGs has previously been shown to induce conformational changes, in contrast to peptide inhibitors, for which no change in secondary structure was determined [47]. In addition, DSF measurements have previously identified a reduction in the melting temperature (T_m_) of BACE1 in the presence of GAGs [9,30], again this is in contrast to other inhibitors for which a stabilising effect has been observed [48]. Here, BACE1 also exhibited a decreased T_m_, when measured using DSF, in the presence of *L. vannamei* F4 in a concentration dependent manner. The reduction in the T_m_ of BACE1 in the presence of *L. vannamei* F4 was two-fold lower than for heparin, comparable to the IC_50_ of *L. vannamei* F4 in FRET-based assays, being double than that of heparin. This provides further support that a reduction in the T_m_ of BACE1 in the presence of GAG-like polysaccharides, when measured using DSF, can infer potential inhibitory activity. The conformational change of BACE1 in the presence of heparin observed by CD experiments, has been shown to results in an increase in α-helix content and reduction in antiparallel β-sheet [9]. This change in secondary structure is in-line with conformational changes observed between BACE1 in active (pH 4.0) and inactive (pH 7.4) conformations [47]. The CD spectra of BACE1 in the presence of *L. vannamei* F4 also exhibited an increase in α-helix content and reduction in antiparallel β-sheet. This suggests that inhibition of BACE1 by GAGs occurs via inducing a conformational change, rendering BACE1 inactive, and that this is distinct from the effects of known peptide inhibitors.

In addition to attenuated anticoagulant activities of non-heparin GAGs, which are known to possess minimal inhibition of the coagulation cascade (with the exception of chemically over sulphated CS; [49], further advantages for utilising non-heparin GAGs as potential therapeutics for AD exist. Notably, CS is widely utilised as a nutraceutical and is licenced as a treatment for osteoarthritis [50] for which no significant side effects have been reported [51]. This is in contrast to some heparin pharmaceuticals, in which heparin induced thrombocytopenia (HIT) is a severe off-target affect. Moreover, non-heparin GAGs have previously been demonstrated to display favourable pharmacokinetics and in addition to subcutaneous injection have been shown to be bioavailable through oral administration [50,51]. This is superior to heparin-like GAGs, which require additional formulations to enable absorption when administered through oral routes and to peptide inhibitors that can display unfavourable pharmacokinetic properties [6]. Chondroitin sulphate, HS and derivatives thereof have also been shown to exert favourable activities against oxidative stress, tau aggregation, the prevention of Aβ associated apoptosis, and possess anti-inflammatory properties [51,52,53,54], demonstrating the potential of non-heparin GAGs as a multi-target drug for the treatment of AD, which would be amenable to oral prophylactic administration.

The use of marine-derived GAGs offers the further advantage of offering increased structural diversity and being easily extractable from aquaculture waste, making this a viable, economically and environmentally attractive source for therapeutic exploitation. 

## 4. Methods

### 4.1. Isolation of Glycosaminoglycans from Litopenaeus vannamei

*L. vannamei* tissue (3.5 kg; Wm Morrisons, Bradford, UK) was defatted by homogenisation in excess acetone (VWR, Lutterworth, UK) and the mixture was incubated for 24 h at room temperature (r.t). Defatted tissue was centrifuged for 10 min at 5670 *g* and the remaining acetone allowed to evaporate overnight. The dried tissue was subsequently digested for 24 h at 60 °C with Alcalase (~17 U.kg^−1^ of dried tissue mass; Novozymes, Bagsvaerd, Denmark) in PBS, 1M NaCl, pH 8.0. Debris was removed via centrifugation for 10 min at 5670 *g*, before addition of Amberlite IRA-900 ion (exchanged into the hydroxide counterion form; Sigma-Aldrich, Dorset, UK) into the supernatant. Following incubation at r.t. for 24 h with agitation, the resin was recovered and washed sequentially with 10 volumes of dH_2_O and 1 M NaCl (r.t.). Bound material was eluted by incubating the resin in 3 M NaCl under agitation for 24 h (r.t). The resin was removed by filtration prior to precipitation of the crude GAG extract with ice cold methonol (1:1 *v*/*v*) at 4 °C for 48 h (VWR, Lutterworth, UK). The crude GAG extract was collected via centrifugation at 15,400 *g* (4°C) for 1 h, resuspended in dH_2_O and dialysed for 48 h against dH_2_O (3.5 kDa MWCO; Biodesign, Carmel, NY, USA). The solution was filtered (0.2 µm syringe filter) and lyophilised prior to resuspension in 1 mL of ddH_2_O (Fisher Scientific, Loughborough, UK). The crude GAG extract was then fractionated by DEAE-Sephacel chromatography, loading onto a pre-packed column (10 mm I.D. × 10 cm; GE Healthcare, Buckinghamshire, UK) at a flow rate of 1 mL × min^−1^. Fractions were eluted with a step-wise gradient of NaCl, with in-line monitoring at 232 nm and 210 nm (not shown) (UV/vis, Cecil Instruments, Cambridge, UK). Six fractions were collected (F1–6) corresponding to 0, 0.25, 0.5, 0.8, 1 and 2 M NaCl, respectively. Each fraction was dialysed for 48 h against dH_2_O, prior to lyophilisation and storage at 4 °C.

### 4.2. Agarose-Based, Gel Electrophoresis

Gel electrophoresis of GAGs (5 μg) was conducted utilising 8 × 8 cm, 1.5 mm thick gels composed of 0.55% (*w*/*v*) agarose in 50 mM 1,3-diaminopropane-acetate buffer, pH 9.0 (VWR, Altrincham, UK). Electrophoresis was performed at 150 V for 30 min in 50 mM 1,3-diaminopropane-acetate buffer using a X-Cell SureLock ™ Mini-Cell Electrophoresis System (ThermoFisher, Altrincham, UK). Subsequent to electrophoretic separation, gels were subjected to cetyltrimethylammonium bromide (CTA) precipitation (0.1% *w*/*v*) for 4 h and dried overnight. Staining was performed in acetic acid:ethanol:H_2_O (0.1:5:5 *v*/*v*) solution supplemented with 0.1% (*w*/*v*) toludine blue for 1 h and de-stained in the same solution without the toludine blue dye. Images were acquired using GIMP software (v2.8, Berkeley, CA, USA) and processed with Image J (v1.51 (100), Madison, QI, USA).

### 4.3. Attenuated Total Reflectance Fourier Transform Infrared Spectroscopy

Attenuated Total Reflectance Fourier Transform Infrared Spectra were recorded using 10 mg of freeze-dried sample on a Bruker Alpha 1 instrument. An average of 32 scans over 5 repeats were carried out with a spectral resolution of 2 cm^−1^ over the range 400–4000 cm^−1^. Spectral acquisition, background correction and data analysis were performed using Opus software (v8.1, Bruker, UK), an Asus Vivibook Pro (M580VD-EB76, Taipei, Taiwan) and R Studio (v1.1.463; Boston, MA, USA). Spectral smoothing (Savitzky-Golay) using a 2nd degree polynomial (21 neighbours) and baseline correction (7th-order polynomial and normalization between 0–1) were applied. The spectral regions between 2000–2500 cm^−1^, below 700 cm^−1^, and above 3600 cm^−1^ were removed to diminish the effects of environmental variations (CO_2_ and H_2_O regions) prior to the plotting of second derivatives (Savitzky–Golay algorithm, 2nd order polynomial with 41 neighbours). Principal component analysis (PCA) was performed on the normalized and corrected matrix of intensities, employing singular value decomposition through R studio (mean-centered, base prcomp function).

### 4.4. Strong Anion-Exchange High Performance Liquid Chromatography HS/Heparin Discacchaide Compositional Analysis

*L. vannamei* samples and a porcine heparin control (50 µg) were subject to exhaustive bacterial lyase digestion by the sequential addition of recombinant heparinase enzymes (I, III and II; 2.5 mIU·mg^−1^; Iduron, Manchester, UK) in 25 mM sodium acetate containing 5 mM calcium acetate at pH 7.0. Samples were incubated at 37 °C for 4 h prior to addition of all three enzyme and incubation at 37 °C, overnight. A ProPac PA-1 analytical column (4 × 250 mm, Dionex, Loughborough, UK), was pre-equilibrated in HPLC-grade H_2_O (1 mL min^−1^) prior to the injection of the digested samples. The flow was held isocratic in H_2_O for 10 min prior to a linear gradient from 0 to 2 M NaCl (HPLC grade; VWR, Lutterworth, UK) over 60 min. Detection of eluted Δ-disaccharides was carried out by monitoring UV absorbance at λ = 232 nm. Retention times were correlated against the 8 common Δ-disaccharide reference standards for heparin according to [40] (Iduron, Manchester, UK). The ProPac PA-1 column was washed with 2 M NaCl and HPLC-grade H_2_O prior to use and between runs. 

### 4.5. Strong Anion-Exchange High Performance Liquid Chromatography Chondrotin Sulphate Discacchaide Compositional Analysis

*L. vannamei* samples, chondrotin sulphate A and chondrotin sulphate C (50 µg) were subject to exhaustive digestion by chondroitin ABCase (Sigma, Dorset, UK) in 50 mM Tris-HCl containing 60 mM sodium acetate and 0.02% *w*/*v* BSA at pH 8. Samples were incubated at 37 °C for 4 h prior to a subsequent addition of further chondroitin ABCase and incubation at 37 °C overnight. A SphereClone analytical column (4.6 × 250 mm, Phenomenex, Macclesfield UK), was pre-equilibrated in HPLC-grade H_2_O, pH 3.5 (1 mL·min^−1^) prior to the injection of the digested samples. The flow was held isocratic in H_2_O for 10 min prior to a linear gradient from 0 to 2 M NaCl, pH 3.5 (HPLC grade; VWR, Lutterworth, UK) over 60 min. Detection of eluted Δ-disaccharides was carried out by monitoring UV absorbance at λ = 232 nm. Retention times were correlated against the 4 most common Δ-disaccharide reference standards for CS according to [40] (Iduron, Manchester, UK). The SphereClone column was washed with 2 M NaCl pH 3.5 and HPLC-grade H_2_O pH 3.5 prior to use and between runs.

### 4.6. Nuclear Magnetic Resonance

*L. vannamei* samples were lyophilised prior to resuspension in D_2_O (600 µL; VWR, Lutterworth, UK); this process was repeated thrice. NMR experiments were conducted at 298 K using an Avance Neo 800 MHz spectrometer equipped with a 5 mm TXI Probe 800 MHz (Bruker, Coventry UK). In addition to 1-dimensional (^1^H) spectra, ^1^H–^13^C Heteronuclear Single-Quantum Correlation (HSQC) 2-dimensional spectra were collected using standard pulse sequences. Spectra were processed and integrated using TopSpin software (4.1.0; Bruker, Coventry, UK) and plotted using Mnova (Mestrelab, Santiago de Compostela, Spain).

### 4.7. Glycosaminoglycans from Litopenaeus vannamei Inhibit β-Secretase 1

Litopenaeus vannamei F4, F5 and heparin were assayed for inhibitory activity against tag-free, human BACE1 (ACRO Biosystems, Newark, Delaware, USA), using the fluorescence resonance energy transfer (FRET) assay. Human BACE1 (312.5 ng) and L. vannamei F4, F5 or heparin were incubated in 50 mM sodium acetate (pH 4.0, 37 °C for 10 min), prior to the addition of a quenched, fluorescent peptide substrate (6.25 μM; MCA-SEVNLDAEFRK(DNP)RR-NH2; Biomatik, Kitchener, ON, Canada) pre-incubated at 37 °C for 10 min in a final well volume of 50 µL. Fluorescent emission was monitored at λ_exc_ = 320 nm, λ_emm_ = 405 nm, over 90 min using a Tecan Infinite^®^ M200 Pro multi-well plate reader (Tecan Group Ltd., Zürich, Switzerland) with i-control™ software. ΔRFU.min^−1^ was calculated through the linear range of the control with no inhibitor, with normalized percentage inhibition calculated (% ± SD, n = 3) compared to the mean of the substrate only and no inhibitor controls, prior to the fitting of a four-parameter logistics model with Prism 7 (GraphPad Software, San Diego, CA, USA).

### 4.8. Activated Partial Thromboplastin Time

Glycosaminoglycan samples (or control), pooled, normal human plasma (citrated; Technoclone, Vienna, Austria) and Pathromtin SL reagent (Siemens, Munich, Germany) were incubated for 120 s (37 °C) prior to the addition of 50 mM CaCl_2_ (VWR, Lutterworth, UK; Vtot = 175 uL, 1:2:2:2 *v*/*v*). Clot formation times were determined using a Thrombotrak Solo coagulometer (Axis-Shield, Dundee, UK). An upper maximal of 120 s, representing 100% inhibition of clotting was adopted. Water (0% clot inhibition, representing a normal aPTT clotting time, of around 37–40 s) and porcine heparin (193 IU.mg^−1^; Celsus, Cincinnati, OH, USA) were used as controls. EC_50_ values were calculated by the fitting of a sigmoidal dose response curve (GraphPad Prism 7, San Diego, CA, USA).

### 4.9. Prothrombin Time

Glycosaminoglycan samples (or control) and pooled, normal human plasma (citrated; Technoclone, Vienna, Austria) were mixed for 60 s (37 °C) prior to the addition of Thromborel S reagent (Siemens, Munich, Germany; Vtot = 150 uL, 1:1:1 *v*/*v*). Clot formation times were determined using a Thrombotrak Solo coagulometer (Axis-Shield, Dundee, UK) with an upper maximal of 2 min (representing 100% inhibition of clotting). H_2_O (0% clot inhibition, representing a normal PT clotting time of around 13–14 s) and porcine heparin (193 IU.mg^−1^; Celsus, OH, USA) were used as controls. EC_50_ values were calculated by the fitting of a sigmoidal dose response curve (GraphPad Prism 7, San Diego, CA, USA).

### 4.10. Differential Scanning Flurimetry

Differential scanning fluorimetry (DSF) was carried out as per the modified method of Niesen et al. (2007) [55], as described by Uniewicz et al. (2010) [56]. Human BACE1 (1 μg), 20× Sypro Orange and heparin, *L. vannamei* GAG or dH_2_O control were assayed by DSF in 50 mM sodium acetate, pH 4.0, in a final well volume of 40 μL using 96-well qPCR plates (AB Biosystems, Warrington, UK) using an AB Biosystems StepOne plus qPCR machine. The TAMRA filter was employed using an initial incubation phase of 20 °C for of 2 min, followed by an increase of 0.5 °C per 30 s up to a T_max_ of 90 °C. Data analysis using Prism 7 (GraphPad Software, San Diego, CA, USA) was carried out, plotting the smoothed (19 neighbours, 2nd-order polynomial; Savitzky-Golay) first derivative. The peak (T_m_) of the first derivative was ascertained using MatLab software (R20018a, MathWorks, Cambridge, UK).

### 4.11. Circular Dichroism

The circular dichroism (CD) spectra of native human BACE1 (6.12 μM, 30 μL; ACRO Biosystems, Newark, Delaware, USA) in 50 mM sodium acetate (pH 4.0; VWR, Lutterworth, UK) was recorded on a J-1500 Jasco CD spectrometer controlled via Spectral Manager II software and calibrated prior to use against (+)-10-camphorsulfonic acid (1 mg·mL^−1^). A 0.2 mm pathlength quartz cuvette (Hellma, Plainview, NY, USA) was employed with a scan speed of 100 nm.min^−1^ and a 1 nm resolution (180–260 nm). All spectra were the mean of five independent scans. BACE1 was buffer exchanged prior to spectral acquisition using a 10-kDa centrifugal filter (Sartorius, Goettingen, Germany) at 12,000 *g* washed three times. Collected data was further process using GraphPad Prism 7 (smoothed to 9 neighbours, 2nd-order polynomial). The prediction of protein secondary structure was obtained using the BeStSel analysis server on unsmoothed data [45]. To ensure the CD spectral changes that were observed when BACE1 was recorded in the presence of each GAG were not an artefact of the addition of the GAG alone (GAGs are known to possess CD spectra at high concentrations [57], GAG control spectra were subtracted before analysis. Additionally, the theoretical, additive CD spectra was verified to differ from the observed CD spectra, indicating that changes in the CD spectra, compared to that of BACE1 alone, are a result of a conformational change upon the interaction with the GAG. The conformational change is thought to occur owing to changes solely in BACE1 secondary structure, as GAG controls exhibited all but negligible spectra when assayed at the concentration used. All CD data presented have had the relevant GAG controls subtracted and have been normalized at 260 nm.

## Figures and Tables

**Table 1:** Disaccharide composition analysis of L. vannamei F4, F5, CSA and CSC. N.D; not detected.

Δ-Disaccharide	*L. vannamei* F4 (%)	*L. vannamei* F5 (%)	CSC (%)	CSA (%)
Δ-UA-GalNAc	18.3	8.8	3.7	2.4
Δ-UA-GalNAc(6S)	75.8	43.0	72.0	4.3
Δ-UA-GalNAc(4S)	48.6	3.4	18.2	93.2
Δ-UA-GalNAc(4S,6S)	*N. D*	44.9	6.0	*N. D*

**Table 2:** Disaccharide composition analysis of *L. vannamei* F4, F5, heparin and HS.

Δ-Disaccharide	*L. vannamei* F4 (%)	*L. vannamei* F5 (%)	Heparin (%)	HS (%)
Δ-UA-GlcNAc	14.8	9.4	8.5	37.7
Δ-UA-GlcNS	9.6	7.6	3.5	17.8
Δ-UA-GlcNAc(6S)	3.7	5.7	5.5	12.8
Δ-UA(2S)-GlcNAc	1.1	0.8	2.8	0.4
Δ-UA-GlcNS(6S)	31.7	47.9	18.2	18.0
Δ-UA(2S)-GlcNS	23.7	12.9	7.6	6.0
Δ-UA(2S)-GlcNAc(6S)	0.5	0.6	2.5	0.4
Δ-UA(2S)-GlcNS(6S)	14.9	15.0	51.4	7.0

**Table 3:** Therapeutic ratio of *L. vannamei* F4 and F5 compared to heparin. The therapeutic ratio was calculated from the IC_50_ of BACE1 inhibitory activity measured by FRET:aPTT anticoagulant activity. GAG = glycosaminoglycan, PMIH = porcine mucosal intestinal heparin, aPTT = activated partial thromboplastin time, PT = prothrombin time.

GAG	aPTT (μg·mL^−1^)	PT (μg·mL^−1^)	BACE1 Inhibitory Activity (μg·mL^−1^)	Therapeutic Ratio
PMIH	1.66	19.53	2.43	0.68
*L.vannamei* (F4)	27.91	1276.00	4.61	6.10
*L.vannamei* (F5)	14.60	138.80	5.93	2.46

**Figure A1:**
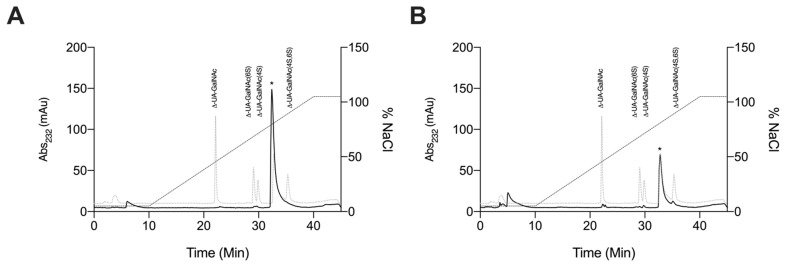
UV-SAX HPLC disaccharide composition analysis was performed on the ABC chondroitinase digest of *P. vannamei* F4 (**A**) and F5 (**B**) ([A]_232 nm_) eluting with a linear gradient of 0–2 M NaCl (dashed line), pH 3.5. Eluted Δ-disaccharides were referenced against four common standards present with CS (light grey dotted line). * Indicates contaminant peaks.

**Figure A2:**
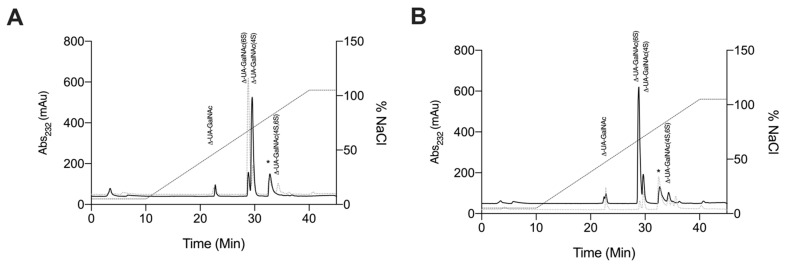
UV-SAX HPLC disaccharide composition analysis was performed on the ABC chondroitinase digest of CSA F4 (**A**) and CSC (**B**) ([A]_232 nm_) eluting with a linear gradient of 0–2 M NaCl (dashed line), pH 3.5. Eluted Δ-disaccharides were referenced against four common standards present with CS (light grey dotted line). * Indicates contaminant peaks.

## Data Availability

The data presented in this study are available within the article and Appendix A.
